# 

**Published:** 1858-03

**Authors:** 


### LIST OF COLLABORATORS.

---

S. G. Armor, M.D., Professor of Pathology and Clinical Medicine, Missouri Med. College.
John L. Atlee, M.D., President Pennsylvania State Medical Society.
Walter F. Atlee, M.D., Philadelphia.
John Bell, M.D., Philadelphia, formerly Professor of Medicine, Medical College of Ohio.
T. S. Bell, M.D., Professor of Medicine, University of Louisville.
S. M. Bemiss, M.D., Louisville, Ky.
L. P. Blackburn, M.D., Natchez, Miss.
George C. Blackie, M.D., Professor of Botany, University of Nashville.
G. C. Blackman, M.D., Professor of Surgery, Medical College of Ohio.
W. S. Bowen, M.D., New York.
J. L. Bobbs, M.D., formerly Professor of Anatomy, Indianapolis.
W. M. Boling, M.D., Montgomery, Ala., formerly Professor of Midwifery, Transylvania University, Lexington, Ky.
N. Bozeman, M.D., Montgomery, Ala.
J. T. Bradford, M.D., Augusta, Ky.
John H. Brinton, M.D., Lecturer on Operative Surgery, Philadelphia.
W. S. Chipley, M.D., Professor of Medicine, Transylvania University.
A. Clapp, M.D., New Albany, Ia.
D. B. Cliffe, M.D., Franklin, Tenn.
J. Da Costa, M.D., Lecturer on the Practice of Medicine at the Philad. Med. Association.
M. Dupierris, M.D., Havana, Cuba.
W. F. Edgar, M.D., United States Army.
S. C. Farrar, M.D., Jackson, Miss.
C. S. Fenner, M.D., Memphis, Tenn.
Austin Flint, M.D., Professor of Clinical Medicine and Pathology, University of Buffalo.
Charles Frick, M.D., Baltimore.
W. Y. Gadbury, M.D., Benton, Miss.
O. C. Gibbs, M.D., Chautauque County, New York.
Dr. J. P. Hall, Glasgow, Ky.
John Hardin, M.D., Professor of Obstetrics, Kentucky School of Medicine, Louisville.

EDWARD HARTSHORNE, M.D., One of the Surgeons to Wills Hospital for Diseases of the Eye, Philadelphia.

B. HASKINS, M.D., Clarksville, Tenn., late President of the Tennessee State Medical Society.

C. A. HENTZ, M.D., Marianna, Florida.

ADDINELL HEWSON, M.D., One of the Surgeons to Wills Hospital for Diseases of the Eye, Philadelphia.

SAMUEL L. HOLLINGSWORTH, M.D., late Editor of the Medical Examiner, Philadelphia.

WILLIAM A. HAMMOND, M.D., United States Army.

WILSON JEWELL, M.D., Philadelphia, President of the Quarantine and Sanitary Convention.

G. A. KUNKLER, M.D., Madison, Ia.

WM. V. KEATING, M.D., Lecturer on Obstetrics and the Diseases of Women, in the Philadelphia Medical Association.

BEN. LOGAN, M.D., Shreveport, La.

A. LOPEZ, M.D., Mobile, Ala., formerly President of the Alabama State Medical Society.

GEORGE R. MOREHOUSE, M.D., Philadelphia.

S. WEIR MITCHELL, M.D., Lecturer on Physiology at the Philad. Med. Association.

B. I. RAPHAEL, M.D., New York.

J. C. REEVE, M.D., Dayton, Ohio.

L. C. RIVES, M.D., formerly Professor of Midwifery, Medical College of Ohio.

J. LAWRENCE SMITH, M.D., Professor of Chemistry, University of Louisville.

W. C. SNEED, M.D., Frankfort, Ky., President Kentucky State Medical Society.

C. H. SPILMAN, M.D., Harrodsburg, Ky., late President of Kentucky State Medical Society.

GEO. SUTTON, M.D., Aurora, Ia.

W. L. SUTTON, M.D., Georgetown, Ky., formerly President Kentucky State Medical Society.

HORATIO R. STORER, M.D., formerly one of the Physicians to the Boston Lying-in Hospital, Boston, Mass.

S. THOMPSON, M.D., Albion, Ill.

E. WILLIAMS, M.D., Cincinnati, Ohio.

### Bi-Monthly Bibliographical Record.

AMERICAN.

Transactions of the Illinois State Medical Society for the year 1857; pp. 128. (Received from committee of publication.)

Discussion on Puerperal Fever, before the New York Academies of Medicine. Remarks by B. F. Barker, M.D., Professor of Obstetrics in the New York Medical College. (Received from the author.)

Nineteenth Annual Report of the Board of Trustees and Officers af the Central Ohio Lunatic Asylum. (Received from Dr. Hills.)

Report of the Board of Managers of the Eastern Kentucky Lunatic Asylum (at Lexington) for 1856–57.

Twenty-First Annual Report of the Vermont Asylum for the Insane.

Biography and Memorial of the late Dr. S. P. Hullihen, of Wheeling, Va. Philadelphia, 1858. (From Dr. E. B. Gardette.)

FOREIGN.

Handbook of the Science and Practice of Medicine. By Wm. Aitken, M.D. Edinburgh, 1857.

A Manual of Medical Diagnosis; being an Analysis of the Signs and Symptoms of Disease. By A. W. Barclay, M.D., F.R.C.P., etc. Reprinted by Blanchard & Lea: Philadelphia, 1858. 8vo., pp. 423.

A treatise on Rheumatic Gout, and Chronic Arthritis of all the Joints. By Robert Adams, M.D., A.M., M.R.J.A., etc. London, 1857; with Atlas of plates.

The Physiology and Treatment of Placenta Previa; being the Lettsomian Lectures on Midwifery for 1857. By Robert Barnes, M.D.; pp. 208. London, 1858.

The Phenomena of Spinal Irritation and other Functional Diseases of the Nervous System. By Thomas Inman, M.D. London, 1857.

The Enlarged Prostate; Its Pathology and Treatment. By Henry Thompson, F.R.C.S. London, 1857.

Prostitution, considered in its Moral, Social, and Sanitary Aspects, in London and other large cities. By Wm. Acton, M.R.C.S. London, 1857.

On the Transmission from Parent to Offspring of some Forms of Disease, and of Morbid Taint and Tendencies. By James Whitehead, M.D.

The Chemistry of Wine. By G. T. Mulder. Edited by H. Bence Jones, M.D. London, 1857; pp. 390.

Tables for the Diagnosis of Diseases of the Brain. By J. Russell Reynolds, M.D. London, 1857; pp. 7.

On Plastic Operations for the Restoration of the Lower Lip. By T. P. Teale, Surgeon to the Leeds General Infirmary. London, 1857.

A Concise History of the Abolition of Mechanical Restraint in the Treatment of the Insane. By R. Gardiner Hill, F.S.A. London, 1857.

Urinary Deposits, their Diagnosis, Pathology, and Therapeutical Indications. By Golding Bird, M.D. Fifth Edition. Edited by E. L. Birkett, M.D. London, 1857; pp. 494.

Contributions to the Physiology and Pathology of the Circulation of the Blood. By George Robinson, M.D. London, 1857.

Human Osteology, comprising a Description of the Bones. By Luther Holden, F.R.C.S. London, 1857; pp. 276.

On Epilepsy and Epileptiform Seizures; their Causes, Pathology, and Treatment. By E. H. Sieveking, M.D. London, 1858; pp. 267.

On the Immediate Principles of Human Excrements in the Healthy State. By W. Marcet, M.D. Trans. Royl. Soc. 1857.

